# Polyethylene microplastic: impacts on ryegrass seed germination and seedling development

**DOI:** 10.1186/s12870-025-06891-2

**Published:** 2025-07-04

**Authors:** Yu Zhang, Xiangtao Wang, Junqin Li, Yuting Yang, Yang Gao, Puchang Wang

**Affiliations:** 1 School of Karst Science, Guizhou Normal University, Guiyang, Guizhou, 550025 China; 2 School of Life Sciences, Guizhou Normal University, Guiyang, Guizhou, 550025 China; 3 School of Life Sciences, Southwest University, Chongqing, 400715 China

**Keywords:** Microplastics, Ryegrass, Seed germination, Seedling growth, Plastic pollution

## Abstract

Microplastic pollution has emerged as a critical global environmental concern, particularly within agricultural ecosystems where its impact on forage production is highly significant. This study used a hydroponic system to investigate the potential effect of polyethylene (PE) microplastics at different concentrations (20, 50, 100, 200, 500, and 1000 mg/L) and particle sizes (200 nm, 25 μm, and 200 μm) on the growth of perennial ryegrass (*Lolium perenne* L.), with no added PE microplastics (0 mg/L) as a control. Our findings indicate that PE microplastics, especially those with a particle size of 200 nm, significantly inhibit ryegrass seed germination. The presence of microplastics disrupts normal water uptake in ryegrass and suppresses biomass accumulation, with the inhibitory effects intensifying as microplastic concentrations increase. Overall, ryegrass seed germination and seedling growth are adversely affected by microplastic exposure levels, with the extent of impact closely associated with both the quantity and size of the microplastics present.

## Introduction

Microplastics, defined as plastic particles with a diameter of less than 5 mm [1], originate either from the fragmentation of larger plastic debris [2, 3] or from microbeads present in certain consumer products, such as facial cleansers and toothpaste [4]. Their small size and ubiquity result in pervasive environmental distribution, spanning from deep-sea habitats to mountainous regions [5, 6], and from urban centers to rural areas [7]. With the escalating production and consumption of plastics, millions of tons of plastic waste enter natural environments annually [8, 9], where they degrade into microplastics through exposure to sunlight, weathering, and biological activity [10, 11], thereby posing significant threats to ecosystems. Microplastics have the capacity to adsorb and transport harmful substances and can traverse the food chain, thereby impacting biodiversity and human health [12]. The Governing Council of the United Nations Environment Programme has identified microplastic pollution as the second most pressing scientific issue in the fields of ecology and environment, underscoring its status as a global environmental challenge [13].

Microplastics are rapidly accumulating in terrestrial and marine ecosystems due to improper waste management and widespread plastic use, adversely affecting plant growth [14, 15]. The impacts of microplastics on plants are primarily twofold [16]. Firstly, microplastics exert direct effects on plants. Microplastic particles may adhere to plant roots, block stomata, and cause mechanical damage to roots, thereby hindering normal plant growth and development [17, 18]. Additionally, microplastics may induce cellular or genetic toxicity in plants [19], adversely affecting their development, metabolism, and nutrient absorption [20]. Secondly, microplastics indirectly impact plants by altering the physical and chemical properties of soil and affecting soil microbial communities [21–23], which in turn influence plant growth.

Research on the effects of microplastics on crop seed germination and growth has become well-established. De Silva et al. [24] were the first to demonstrate the inhibitory effect of microplastics on crop seed germination by studying polyethylene (PE) microplastics'effects on lentil seed germination and growth, primarily attributing the inhibition to the physical blockage of stomata by microplastics. Guo et al. [25] observed that increasing concentrations of various types of polystyrene microplastics significantly decreased the germination rates of three herbaceous plant species. Studies on the impact of microplastics on the germination and growth of crop seeds have predominantly focused on wheat [26], soybeans [27], corn [28], with relatively limited research on the important forage crop ryegrass. Polyethylene (PE), one of the most prevalent plastics, is extensively used in packaging materials, plastic bags, agricultural films etc. [29]. Its chemical and abrasion resistance, coupled with low cost, has led to widespread usage across various sectors [30]. Consequently, PE microplastics are particularly prevalent in the environment, found globally in oceans, soils, rivers, and lakes, with their ecological impact becoming increasingly apparent [31]. One of the main sources of microplastics in agricultural ecosystems is plastic film and greenhouse film, mainly composed of PE as the main polymer [32]. They are difficult to recycle and usually remain in the soil, gradually degrading into microplastics over time. This leads to contamination of agricultural soils, adversely affecting crop growth and food security.

Forage growth is fundamental to animal husbandry, intricately linked to animal health and productivity. Microplastic pollution's impact on forage growth is not only crucial for the sustainable development of agriculture but may also have implications for human health through the food chain. Ryegrass, a vital forage crop [33], serves as a benchmark for assessing the impact of microplastics on forage crops. Investigating the effects of microplastics on ryegrass seed germination and seedling growth is essential for understanding and mitigating the potential risks of microplastic pollution in agroecosystems.

The aim of this study was to investigate the effects of PE microplastics of different particle sizes and concentrations on ryegrass seed germination and seedling growth. The impact of microplastics on ryegrass seed germination rate was assessed by varying the concentration of microplastics under controlled experimental conditions. Additionally, the effects of microplastics on ryegrass seedling growth were evaluated by measuring parameters such as seedling height, root length, dry weight, and fresh weight. Through these investigations, this study aims to elucidate the potential effects of microplastic pollution on the growth of fodder crops and to inform the development of strategies to mitigate microplastic pollution.

## Materials and methods

### Materials for testing

Perennial ryegrass (*Lolium perenne* L.) seeds were obtained from Bailiwick Green Forage Co. Tween 20 was used as a surfactant. Referring to relevant literature on the effects of microplastics on seed germination [34–36], this study selected three different particle sizes of microplastic powders, namely 200 nm, 25 μm, and 200 μm, respectively. The microplastic powders were purchased from Jiangsu Zhongcai Tetrafluor New Materials Co., Ltd., and Guangdong Dongguan Qianjing New Materials Co., Ltd.

### Microplastic concentration setting and suspension preparation

Drawing on the concentration settings from the experiments conducted by Zhu et al. [37] and Murphy et al. [38], we selected microplastic concentrations of 20, 50, 100, 200, 500, and 1000 mg/L for this study. The preparation procedure involved accurately weighing 20, 50, 100, 200, 500, and 1000 mg of each microplastic powder and adding the respective amounts to 1 L of ultrapure water, control group without adding PE powder. Subsequently, 0.1% (v/v) Tween 20 was added to each mixture. The mixtures were then homogenized using a high-speed homogenizer (FSH-2A) for 30 min to ensure a uniformly dispersed microplastic suspension at the specified concentrations.

### Seed germination experiment

Two sheets of qualitative filter paper were placed in 90 mm glass Petri dishes and moistened with ultrapure water to eliminate any air bubbles beneath the filter paper. The ryegrass seeds were sterilized by soaking in a 5% NaClO solution for 5 min, followed by thorough rinsing with ultrapure water. Subsequently, 30 sterilized seeds were evenly distributed in each prepared Petri dish, with four replicates per treatment. To each dish, 5 mL of microplastic suspension at concentrations of 20, 50, 100, 200, 500, and 1000 mg/L were added. Control dishes were treated with deionized water containing 0.1% (v/v) Tween 20. The Petri dishes were then placed in an artificial climate incubator set to a light intensity of 5500 lx, humidity of 50%, and a temperature of 25 °C for a duration of 14 days (from May 4, 2024, to May 17, 2024). Ryegrass seed germination was recorded daily throughout the incubation period. Starting on the third day, the Petri dishes were briefly opened and supplemented with 1–2 mL of ultrapure water to compensate for any evaporated moisture. Record the number of sprouts when the embryonic root length is ≥ 2 mm.

The control group in this study strictly followed OECD Guideline 208: The standard requirements of the Territorial Plant Test (hereinafter referred to as OECD 208) [39]. OECD 208, as an internationally recognized ecotoxicological testing guideline, aims to systematically evaluate the potential effects of chemicals on seed germination and early seedling growth stages of terrestrial plants. This standard stipulates that the germination rate of the ryegrass control group must be ≥ 70% (with the criterion of the embryo root breaking through the seed coat ≥ 2 mm), and growth parameters such as root length must be quantitatively recorded. Although the guideline does not set an absolute limit on the root length of the control group, based on literature reports [40–42], the typical root length range of perennial ryegrass under standard test conditions (10–14 days cultivation period) is 3–8 cm. To ensure the reliability of the experimental data, this study specifically set the acceptable threshold for the root length of the control group to ≥ 3 cm.

### Measurement methods

On the 14th day of incubation, seedlings were harvested for measurement. Seedling length and root length were measured using a Vernier caliper, while fresh weight was determined using an analytical balance. The ryegrass seedlings were then dried in an oven at 105 °C for 24 h to achieve a constant mass, after which dry weight was recorded using the balance. The following formulas were used to calculate the germination rate, germination potential, germination index, vitality index, and inhibition rate:

1 $$\begin{array}{c}\text{Germination Energy}=\frac{\text{Number of seeds germinated in the first 3 days}}{\text{Total number of test seeds}}\times 100 \%\end{array}$$

2 $$\begin{array}{c}\text{Germination Rate}=\frac{\text{The number of germinated seeds of the end of germination}}{\text{Total number of test seeds}}\times 100 \%\end{array}$$

3 $$\begin{array}{c}\text{Germination Index}=\sum \frac{\text{Number of germinated seeds on day t}}{\text{Corresponding germination days}}\end{array}$$

4 $$\begin{array}{c}\text{Vitality Index=Germination Index}\times \text{Dry weight}\end{array}$$

5 $$\begin{array}{c}\text{Germination inhibition rate}\\ =\frac{\left(\text{ Seeds germination rate of control group}-\text{Seeds germination rate of treat group }\right)}{\text{Seeds germination rate of control group}}\times 100\%\end{array}$$

### Data processing

Experimental results were expressed as mean ± standard error (Mean ± SE). Data were processed using Microsoft Excel 2019, statistically analyzed with SPSS 27.0, and figures were generated using Origin 2022. One-way analysis of variance (ANOVA) was performed, followed by Duncan's post-hoc test to determine statistical differences among treatments. Statistical significance was set at *P* < 0.05. In cases where variances were unequal, if the data passed Welch's test, the Tamhane's T2 post-hoc test was employed.

## Results and analysis

### Effect of PE microplastics on germination rates

**Fig. 1:**
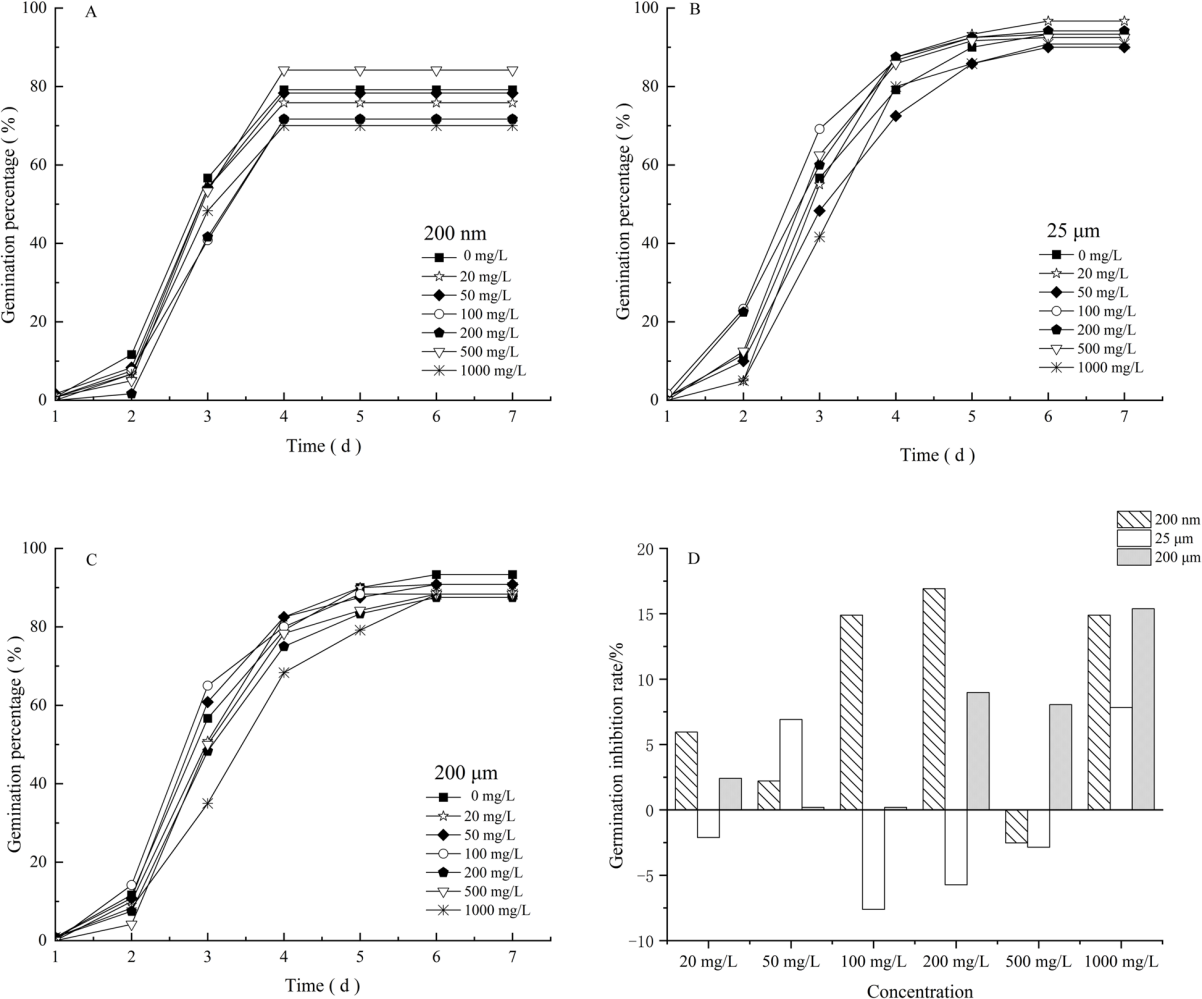
Germination and germination inhibition rates of ryegrass seeds. A-C represents the seed germination rate, and D represents the germination inhibition rate. Note: After the 7th day, the number of seeds germinated no longer changed, so the calculation period for the germination rate was the first 7 days

The germination rate serves as a critical indicator of seed vigor under microplastic exposure. The germination percentage of ryegrass seeds exposed to microplastics ranged from 85.8% to 95% (Fig. 1A-C), indicating that the majority of ryegrass seeds were capable of germination under microplastic stress. Both small (200 nm) and large (200 μm) particle-sized microplastics significantly inhibited ryegrass germination across different concentrations (Fig. 1D), with inhibition rates ranging from 2.5% to 17.5%. The inhibitory effect was more pronounced for 200 nm. Additionally, the inhibition increased with microplastic concentration, being more substantial at medium (100 mg/L and 200 mg/L) and high concentrations (1000 mg/L) compared to low concentrations (20 mg/L and 50 mg/L) for both 200 nm and 200 μm. In contrast, medium-sized microplastics (25 μm) predominantly promoted seed germination, with the highest promotion observed at medium concentrations (100 mg/L and 200 mg/L).

### Effect of PE microplastics on the growth characteristics of ryegrass seeds

Microplastics influenced the germination energy, germination index, and vitality index of ryegrass seeds, primarily through the inhibition by small particle sizes. The effects of medium and large particle sizes were concentration-dependent, though the differences were not statistically significant (*P* > 0.05) compared to the control group (Table 1). Specifically, the 200 nm microplastic treatment reduced the germination energy of ryegrass, reaching the lowest value at a concentration of 100 mg/L. For the 25 μm microplastics, germination energy was inhibited at low concentrations and promoted at medium concentrations. At 200 μm, germination energy increased at exposure concentrations of 50 mg/L and 100 mg/L but decreased at higher concentrations. Both 200 nm and 200 μm microplastic treatments decreased the germination index, with the lowest values observed at 100 mg/L and 200 mg/L for the 200 nm particles. The vitality index was generally suppressed by the 200 nm microplastics, while the 25 μm microplastics had variable effects. Specifically, the vitality index increased at medium concentrations (100 mg/L) for the 25 μm particles and was promoted by low and medium concentrations but inhibited by high concentrations in the 200 nm treatments.

**Table 1:** Effects of PE microplastic size and concentration on the growth characteristics of ryegrass seeds

size	concentration(mg/L)	germination energy	germination index	vitality index
	0	56.67 ± 5.93a	3.29 ± 0.20a	0.026 ± 0.002ab
200 nm	20	54.17 ± 3.94a	3.13 ± 0.12a	0.020 ± 0.001a
	50	54.17 ± 4.59a	3.27 ± 0.14a	0.026 ± 0.003a
	100	40.83 ± 0.83a	2.90 ± 0.11a	0.023 ± 0.001a
	200	41.67 ± 7.76a	2.90 ± 0.10a	0.021 ± 0.003a
	500	53.33 ± 3.60a	3.26 ± 0.10a	0.026 ± 0.002a
	1000	48.33 ± 7.01a	3.00 ± 0.10a	0.020 ± 0.002a
25 μm	20	55.00 ± 5.85a	3.32 ± 0.10a	0.029 ± 0.001ab
	50	48.33 ± 6.16a	3.09 ± 0.04a	0.027 ± 0.001b
	100	69.17 ± 5.51a	3.58 ± 0.09a	0.036 ± 0.002a
	200	60.00 ± 5.27a	3.49 ± 0.12a	0.032 ± 0.003ab
	500	62.50 ± 7.98a	3.35 ± 0.21a	0.033 ± 0.002ab
	1000	41.67 ± 9.08a	3.03 ± 0.16a	0.027 ± 0.004b
200 μm	20	50.83 ± 8.96a	3.18 ± 0.19a	0.029 ± 0.003a
	50	60.83 ± 2.10a	3.27 ± 0.09a	0.027 ± 0.002a
	100	65.00 ± 7.26a	3.26 ± 0.15a	0.034 ± 0.004a
	200	48.33 ± 7.76a	3.02 ± 0.13a	0.027 ± 0.003a
	500	50.00 ± 8.28a	3.01 ± 0.13a	0.021 ± 0.002a
	1000	35.00 ± 5.69a	2.90 ± 0.13a	0.025 ± 0.004a

Different letters after the same number indicate significant differences between different treatment groups (*P* < 0.05)

### Effects of PE microplastics on ryegrass seedling length, root length and seedling biomass

**Fig. 2:**
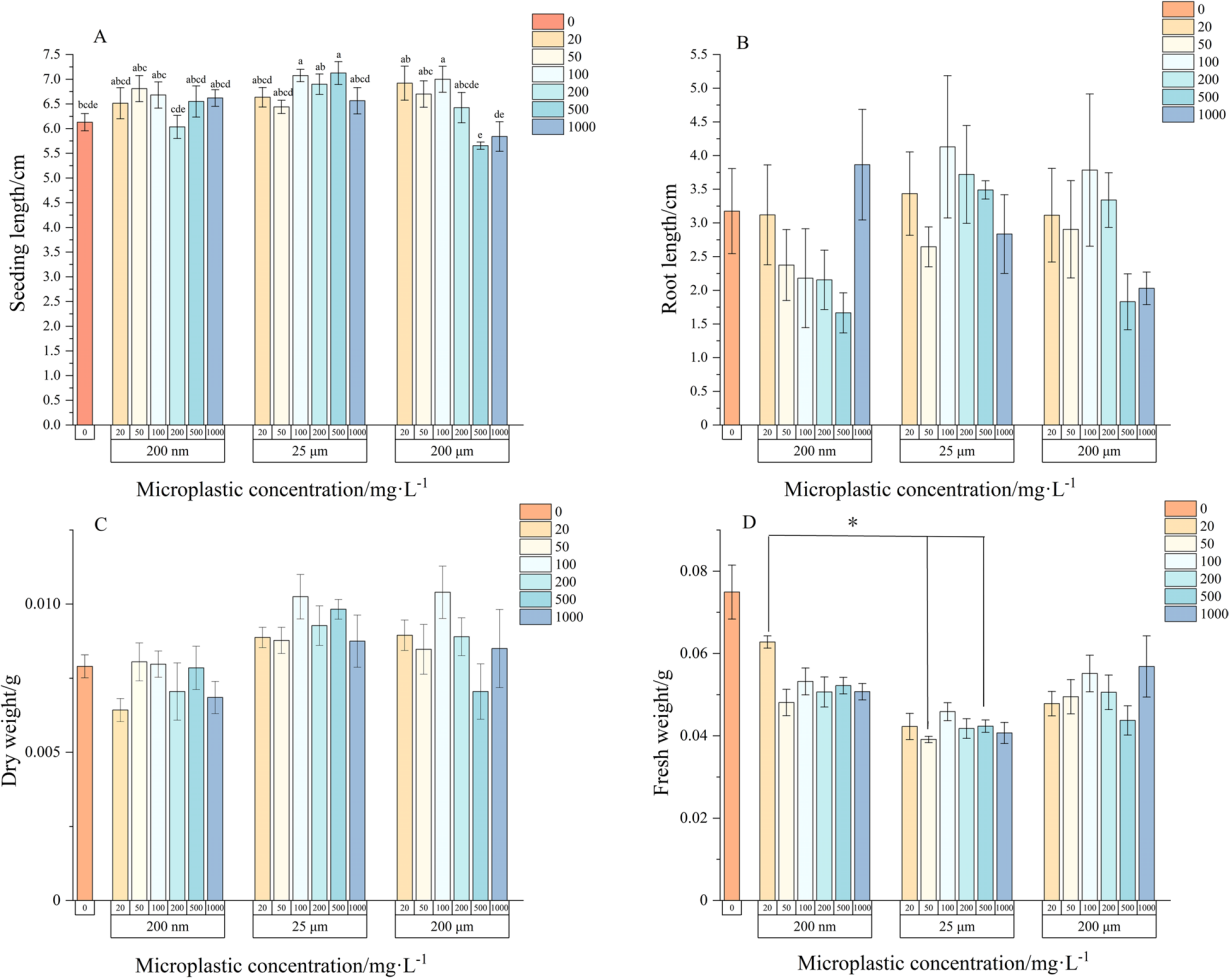
The effect of microplastics on morphological indicators of ryegrass seedlings. A represents the height of the seedlings; B represents root length; C represents dry weight; D represents fresh weight. Note: The lowercase letters in Fig. 2 A and the * in Fig. 2D indicate significant differences between different treatments (*P* < 0.05)

Ryegrass seedling length exhibited significant differences (*P* < 0.05) under different particle size treatments compared to the control (Fig. 2A). Specifically, seedling height was inhibited by the small particle size (200 nm) microplastics at an exposure concentration of 200 mg/L, whereas other treatments promoted seedling elongation. Exposure to 25 μm microplastics consistently promoted ryegrass seedling length, resulting in increases ranging from 5 to 17% compared to the control. In the 200 μm microplastic treatment, seedling growth was enhanced at low to medium concentrations but inhibited at high concentrations, with the most substantial inhibition observed at 500 mg/L.

Ryegrass root length under microplastic exposure did not differ significantly from the control group (*P* > 0.05) (Fig. 2B), suggesting minimal impact of microplastics on root elongation. However, a trend was observed where root length tended to decrease with increasing concentrations of small-sized microplastics, except at the highest concentration (1000 mg/L), which promoted root elongation. For medium-sized microplastics, exposure concentrations of 50 mg/L and 1000 mg/L inhibited root elongation by 16.7% and 11%, respectively, while other concentrations had no significant effect. Large particle-sized (200 μm) microplastics mirrored the effects observed in seedling elongation, promoting root growth at low to medium concentrations and inhibiting it at high concentrations.

Similar to root length, the dry weight of ryegrass seedlings was not significantly affected by microplastic exposure (*P* > 0.05) (Fig. 2C). However, an increase in dry weight was observed under various concentrations of 25 μm microplastics. In contrast, the fresh weight of ryegrass was predominantly inhibited by microplastic exposure (Fig. 2D), with reductions ranging from 39 to 48% under 25 μm microplastics compared to the control. Among all treatments, significant differences (*P* < 0.05) were noted between the 20 mg/L treatment in the 200 nm microplastic environment and the 50 mg/L and 500 mg/L treatments in the 25 μm microplastic environment.

## Discussion

Microplastics that enter agroecosystems persist and accumulate in the soil, potentially disrupting the balance of entire ecosystems by altering soil physicochemical properties [43, 44], and affecting plant and animal growth [45, 46]. Seed germination is a key stage in the plant life cycle and is widely used as an indicator to assess the phytotoxicity of toxic chemicals [47]. The effect of chemicals on seed germination depends on various factors, including the type of chemical, particle size, and plant species [48]. In this study, we observed that smaller particle size (200 nm) microplastics inhibited ryegrass seed germination more significantly, in contrast to 25 μm microplastics that promoted ryegrass seed germination (Fig. 1D, Table 1). This phenomenon may be related to the surface area to volume ratio of microplastics: smaller particles have a larger specific surface area and are more likely to attach to the seed surface by physical adsorption or passive diffusion [17, 49, 50]. This adsorption may affect seed germination through the following mechanisms: (1) blockage of micropores in the seed coat, preventing water and oxygen penetration; (2) induction of reactive oxygen species production, leading to cell membrane damage and inhibition of enzyme activities [51]; and (3) interference with energy metabolic processes, such as respiration and ATP synthesis [52]. Future studies need to combine microscopic observations and molecular biology techniques to further reveal the specific mechanisms of microplastics on seed germination.

In addition, in this study we found that the concentration of microplastics in the environment affects the utilization of water by ryegrass (Fig. 2C-D), a process that affects seed germination as well as later seedling growth processes in ryegrass. Previous studies have also demonstrated that concentration has a significant effect on plant growth and development. For instance, Lian et al. [53] found that microplastics significantly inhibited the germination and growth of wheat seeds, particularly reducing root length and shoot elongation under 10 mg/L treatments. Similarly, Xu et al. [49] reported that plastic mulch adversely affected wheat germination, resulting in significant inhibition of shoot and root elongation at 5000 mg/kg and 15,000 mg/kg concentrations. However, the inhibitory effects of PE microplastics of different particle sizes and concentrations are likely the result of multiple subtle changes in the microplastics themselves, such as “compound biotoxicity”, “physical properties”, and “environmental factors” [54]. Compound biotoxicity means that microplastics in the environment may release chemical additives (e.g., plasticizers, stabilizers) [55] or adsorb other pollutants (e.g., heavy metals, organic pollutants) in the environment [56, 57], which may affect plant growth and development by inducing oxidative stress or interfering with energy metabolism. Some current studies have also focused on the growth response of plants under the coexistence of microplastics and other environmental pollutants; Physical properties are mainly related to the particle size, shape, and surface properties of microplastics [58]. Small particle size microplastics (e.g., 200 nm microplastics in this study) are more likely to adsorb to seed surfaces due to their large variable area and volume ratios, potentially blocking seed coat pores and affecting water and oxygen penetration [59]. Environmental factors (e.g., temperature, humidity, soil pH) may further modulate the toxic effects of microplastics [60]. For example, high ambient temperatures may accelerate the aging of microplastics and increase the release of chemical additives [61]. The specific mechanisms underlying these effects require further investigation.

Microplastics also impact the physiological and biochemical characteristics of plants, primarily affecting the activities of antioxidant enzymes, photosynthetic pigments, and soluble proteins [62, 63]. Photosynthetic pigments are essential components of photosynthesis, and changes in their content directly influence the photosynthetic capacity of plants [64, 65], thereby affecting biomass formation. In this study, we found that microplastics had a significant negative effect on the growth of ryegrass seedlings, with variations observed based on particle size and concentration. The impact of microplastics became more pronounced with increasing concentrations, and 200 nm had a more significant effect than 200 μm, influencing both root growth and changes in dry and fresh weight (Fig. 2). Although the pore size of the cell wall typically ranges between 3.5–5.0 nm [1], submicron-sized microplastics (0.2 μm) may still enter plant tissues [66]. This phenomenon likely occurs due to incomplete development of the Casparian strip in root tips, which can lead to the formation of small fissures [67]. Under transpiration pull, microplastic particles or beads may infiltrate through these fissures, subsequently affecting plant growth and development [66]. Additionally, microplastics can disrupt normal physiological processes through three potential mechanisms: (1) mechanical blockage, (2) seed surface adsorption, and (3) root system interference [68].

Larger particles of microplastics may accumulate on the surface of the root system or in the cell interstitial space, leading to mechanical blockage, which may affect water and nutrient uptake. For example, microplastic particles adhering to the root surface may prevent water infiltration, leading to plant dehydration [17, 24]. In contrast, microplastics attached to the seed epidermis through physical adsorption can affect water uptake and gas exchange of the seed, and this adsorption may lead to delayed or inhibited seed germination [29]. At the same time, larger particles of microplastics may cause mechanical damage to the root system through physical friction or penetration, thus affecting the normal functioning of the root system [62]. Kalcíková, G et al. [69] demonstrated that polyethylene microbeads ranging from 30 to 600 μm significantly affected the root growth of floating ryegrass by causing mechanical blockage. Although we observed an inhibitory effect of PE microplastics on the growth of ryegrass seedlings, the unclear trend in ryegrass response to stress at 25 μm and different concentrations may result from the following reasons: (1) Microplastics with different particle sizes and concentrations may be unevenly distributed in the experimental medium, resulting in local concentration differences; (2) Plant response to microplastics may involve multiple mechanisms (e.g., physical action, oxidative stress), and the interaction of these mechanisms may lead to inconsistent results [26, 70]; (3) Differences between laboratory conditions and the natural environment may affect the actual effects of microplastics.

Moisture is a crucial component involved in photosynthesis. Excluding the consideration of plant root cell pore sizes, we propose two hypotheses to explain the significant reduction in the fresh weight of ryegrass observed in our study (Fig. 2D). First, microplastics may inhibit water uptake by ryegrass through mechanical effects such as adsorption or blockage, thereby reducing the water available for photosynthesis and subsequently inhibiting photosynthetic activity [71, 72]. This reduction in photosynthesis could lead to decreased biomass accumulation. Second, smaller microplastic particles may be directly absorbed by the plant or enter the plant during cell division [73]. The presence of microplastics within the plant could lead to a decrease in chlorophyll a and b content, thereby reducing the photosynthetic rate and ultimately affecting plant biomass [74]. Previous research indicates that PE can diminish various physiological aspects of lettuce plants, including photosynthetic rate, stomatal conductance, instantaneous transpiration rate, fluorescence parameters, and leaf chlorophyll content, primarily due to reduced chlorophyll synthesis and photosynthetic efficiency [75–77]. Although our study did not measure chlorophyll content in ryegrass under microplastic stress, it is evident that PE microplastics adversely affect the water utilization efficiency of ryegrass. It is worth noting that different plants have different responses to microplastic pollution, which may be related to the plant species'and the plant's tolerance to environmental stress. For example, the root structure and cell ratio composition of ryegrass (Poaceae) are significantly different from lettuce (dicotyledonous plant) [74] and soybean (Leguminous plant) [78] and their response to microplastic pollution is also different. Therefore, future comparisons of the response of different plants to microplastic contamination are needed to comprehensively improve the assessment of the potential risk of microplastics in agroecosystems.

In summary, the presence of microplastics affects both seed germination and seedling growth in ryegrass, with more pronounced effects observed at the seedling stage. These effects are contingent upon the particle size and concentration of the microplastics. Future research should focus on the flow and transportation of microplastics within plants, the differential effects on various plant parts, and the impacts across different growth stages. Such studies will provide a more comprehensive understanding of plant responses to microplastic pollution and aid in developing strategies to mitigate its adverse effects on agroecosystems.

## Conclusion

This study reveals that PE microplastics exert differential effects on ryegrass (*Lolium perenne* L*.*) germination and seedling development based on particle size and concentration. Although PE microplastics did not significantly impact the overall germination rate of ryegrass, an inhibitory effect was observed specifically with the smallest particle size (200 nm). More prominently, the presence of PE microplastics significantly hindered ryegrass seedling growth, particularly affecting biomass accumulation. Additionally, microplastic exposure adversely influenced the water utilization efficiency of ryegrass, which may further compromise plant health and productivity. These findings underscore the potential risks that microplastic pollution poses to forage crop development and highlight the need for effective strategies to mitigate microplastic contamination in agricultural ecosystems. Future research should focus on elucidating the underlying mechanisms of microplastic-plant interactions and exploring the long-term implications of microplastic accumulation on crop performance and ecosystem sustainability.

## Data Availability

The date that supports the findings of this study are available from the corresponding author (wangpuchang@163.com) upon reasonable request.
